# Estimating synaptic connections from multiple spike trains based on a coupled escape rate model

**DOI:** 10.1186/1471-2202-14-S1-P213

**Published:** 2013-07-08

**Authors:** Ryota Kobayashi, Katsunori Kitano

**Affiliations:** 1 Department of Human and Computer Intelligence, Ritsumeikan University, Kusatsu, Shiga 5258577, Japan

## 

An approach to study the mechanism of information processing in a neural circuit is to identify synaptic connectivity between neurons. As recent advances in experimental techniques facilitate simultaneous recording of a large population of neurons, estimation of synaptic connectivity from multiple neural spike train data has become a major goal in computational neuroscience. Although it is important to develop an accurate method, it is difficult to validate a method on the basis of experimental data because of the lack of knowledge regarding synaptic connectivity.

In this contribution, we have developed a method to estimate synaptic connection on the basis of a coupled escape rate model (CERM), which is an extension of the escape rate model [1]. The strength of the synaptic connection was determined by maximizing the likelihood function. We applied this method as well as model-free methods (transfer entropy and cross-correlation [2]) to synthetic spike train data. The synthetic data were generated by a cortical network model, which consisted of thousands of Hodgkin-Huxley type neurons [3]. These methods are applied to the spike data generated by the cortical network model with different topologies of synaptic connectivity (regular, small-world, and random). It should be noted that the true connectivity is known and the estimation performance can be evaluated by the receiver operating characteristic (ROC) analysis.

Our results demonstrated that all methods gave better results for nonrandom (regular and small-world) networks than for random networks. Furthermore, CERM is considered to be the optimal method because it does not involve binning and requires smallest observation length. Therefore, despite high computational costs, CERM is a most suitable method to estimate synaptic connections from multiple spike train data.
